# Single Particle Dynamic Imaging and Fe^3+^ Sensing with Bright Carbon Dots Derived from Bovine Serum Albumin Proteins

**DOI:** 10.1038/srep17727

**Published:** 2015-12-04

**Authors:** Qingxiu Yang, Lin Wei, Xuanfang Zheng, Lehui Xiao

**Affiliations:** 1 Dynamic Optical Microscopic Imaging Laboratory, Hunan Normal University, Changsha, Hunan, 410081, P.R. China; 2 Key Laboratory of Chemical Biology & Traditional Chinese Medicine Research, Ministry of Education, Hunan Normal University, Changsha, Hunan, 410081, P.R. China; 3 Key Laboratory of Phytochemical R&D of Hunan Province, College of Chemistry and Chemical Engineering, Hunan Normal University, Changsha, Hunan, 410081, P.R. China

## Abstract

In this work, we demonstrated a convenient and green strategy for the synthesis of highly luminescent and water-soluble carbon dots (Cdots) by carbonizing carbon precursors, i.e., Bovine serum albumin (BSA) nanoparticles, in water solution. Without post surface modification, the as-synthesized Cdots exhibit fluorescence quantum yield (Q.Y.) as high as 34.8% and display superior colloidal stability not only in concentrated salt solutions (e.g. 2 M KCl) but also in a wide range of pH solutions. According to the FT-IR measurements, the Cdots contain many carboxyl groups, providing a versatile route for further chemical and biological functionalization. Through conjugation of Cdots with the transacting activator of transcription (TAT) peptide (a kind of cell penetration peptide (CPP)) derived from human immunodeficiency virus (HIV), it is possible to directly monitor the dynamic interactions of CPP with living cell membrane at single particle level. Furthermore, these Cdots also exhibit a dosage-dependent selectivity toward Fe^3+^ among other metal ions, including K^+^, Na^+^, Mg^2+^, Hg^2+^, Co^2+^, Cu^2+^, Pb^2+^ and Al^3+^. We believed that the Cdots prepared by this strategy would display promising applications in various areas, including analytical chemistry, nanomedicine, biochemistry and so on.

Development of fluorescent materials for biosensing as well as cellular imaging has aroused great attention in recent years due to the exceptional advantages such as better detection sensitivity and easy differentiation of background noise in complex surroundings^1^. Organic fluorescent dye is the most commonly adopted fluorescent contrast reagent so far. They typically exhibit high fluorescence Q.Y. and adjustable emission wavelength in a broad wavelength range. Through conjugation with functional molecules, it is possible to specifically distinguish target objects in a highly efficient way because of their small size dimension. However, the optical absorption cross-section of these materials is normally very small (around 10^−16^ cm^2^)^2^,^3^. It is thus a great challenge to realize highly sensitive detection (i.e. at single molecule (or particle) level) for routine assays. Another limitation is irreversible photobleaching, which greatly limits the applications for long-term dynamic observation especially in living cell system.

The discovery of semiconductor nanocrystals with physical dimension smaller than the exciton Bohr radius opens up a new avenue for fluorescence-based assays^4^,^5^. Owing to the quantum confinement effect, these quantum-sized nanoparticles display systematically predictable dependence of optical properties, especially fluorescence emission colors, on the nanoparticle dimension. Because of the greatly improved fluorescence brightness over organic dye, they have been widely pursued as excellent fluorescent markers for biosensing, imaging and medical diagnosis^6^,^7^,^8^. Since these conventional semiconductor nanocrystals typically contain toxic heavy metal elements such as cadmium or selenium, many concerns have been drawn with respect to their *in-vivo* or even *in-vitro* usage^9^,^10^,^11^,^12^.

On this account, great efforts have been devoted to develop small size and more biocompatible fluorescent nanomaterials^13^,^14^,^15^,^16^,^17^,^18^,^19^,^20^,^21^,^22^,^23^. Among the current promising additions to the quantum-sized nanomaterials, carbon-based nanoparticles (carbon dots or Cdots, with size below 10 nm) are pursed as nontoxic alternatives for fluorescence related assays and biomedical purposes^15^,^16^,^19^. The Cdots are generally composed of sp^2^ hybridized carbon atoms with abundant oxygen- and hydrogen-containing residues on the surface. They exhibit similarly bright fluorescence as conventional semiconducting quantum dots. Compared with organic fluorescent dyes, Cdots have improved photostability, low cytotoxicity and resistance to environmental change. As a consequence of these attractive merits, intense interests have been drawn in bioimaging, photocatalysis, light-emitting devices, optoelectronics and thermal theranostics^24^,^25^,^26^,^27^,^28^,^29^,^30^,^31^,^32^,^33^.

Following the discovery of Cdots by Xu *et al.* in 2004^15^, so far, many interesting methods have been developed to synthesize Cdots, including top-down (such as arc discharge, laser ablation, electrochemical/chemical oxidation) and bottom-up methods (such as carbonizing polymerized resols, dehydration of carbonhydrates using concentrated sulfuric acid)^15^,^16^,^34^,^35^,^36^,^37^,^38^,^39^,^40^,^41^. Typically, these fabrication strategies involve harsh reaction conditions and expensive equipment. The photo-luminescence Q.Y. of those Cdots is commonly very low (typically less than 10%)^16^,^19^,^42^. In view of the significant potential of this zero-dimension carbon nanomaterial in various fields, a moderate, facile, cheap, and fast synthetic route to give bright fluorescence Cdots is highly desired.

In this work, we proposed a convenient and green strategy for the synthesis of highly luminescent and water-soluble Cdots by carbonizing carbon precursors, i.e., BSA nanoparticles, in hot water solution. The obtained water-soluble Cdots have diameter around 2.2 ± 0.4 nm (measured by transmission electron microscopy) and exhibit relative fluorescence Q.Y. of 34.8% with maximum excitation at 405 nm. Fourier transform infrared spectroscopy (FT-IR) measurements illustrate that the surface of Cdots is coated with abundant of carboxyl groups, providing a versatile route for further chemical and biological functionalization. These Cdots display superior colloidal stability not only in concentrated salt solutions (e.g. 2 M KCl) but also in a broad range of pH solutions. Through conjugation of Cdots with functional biomolecules, e.g., CPP, it is possible to directly monitor the dynamic interactions of CPP with living cell membrane. Under similar imaging condition, the signal-to-noise ratio of these Cdots for single particle imaging is comparable with that of commonly used semiconducting quantum dots. More interestingly, these Cdots also display a dosage-dependent selectivity toward Fe^3+^ among other metal ions, including K^+^, Na^+^, Mg^2+^, Hg^2+^, Co^2+^, Cu^2+^, Pb^2+^ and Al^3+^. Therefore, it could be further applied as a Fe^3+^ sensor for real sample analysis in the future.

## Results and Discussion

### Fabrication of BSA Cdots

The commonly adopted approaches for Cdots fabrication (e.g. laser ablation, electrochemical oxidization, chemical oxidation, pyrolysis and so on) are usually involving in complicated experimental procedures as well as harsh reaction conditions^15^,^16^,^34^,^35^. The observed fluorescence Q.Y. of those Cdots is normally very low. To improve the fluorescence brightness, the precursor carbon nanoparticles typically need to be treated with nitric acid for the introduction of functional groups subsequently used for the surface functionalization chemistry. The functionalization reagents included oligomeric polyethylene glycol diamine (PEG1500n) and amino-polymer (PPEI-EI)^16^,^42^. Even though the resulting Cdots exhibited fluorescence Q.Y. up to 10% or slightly higher, the hydrodynamic radius would be unavoidably increased to tens of nanometers, which thus forms a grand challenge for biological labeling applications.

**Figure 1:**
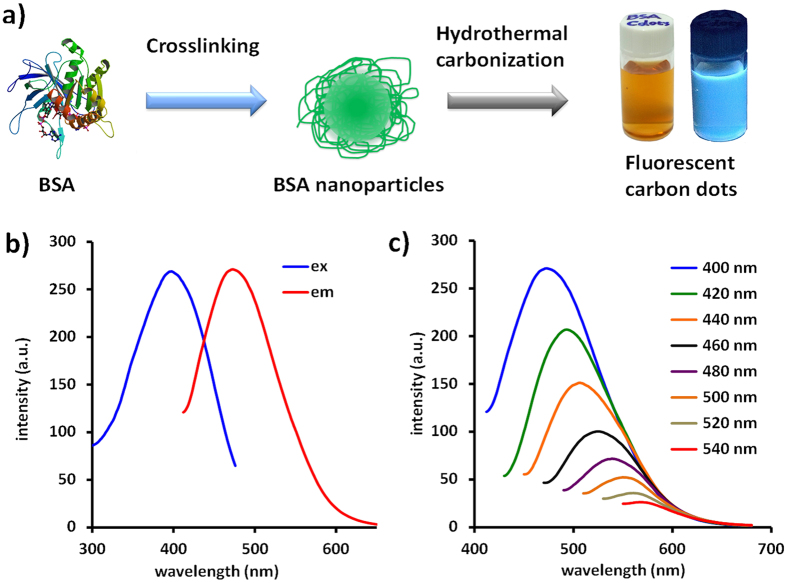
(**a**) Schematic diagram of the procedure for BSA Cdots fabrication. Inserted pictures are the color (left) and fluorescence (right) image of Cdots solution. (**b**) The fluorescence excitation (blue) and emission (red) spectra of BSA Cdots. (**c**) Excitation dependent emission spectra of Cdots.

Herein, we introduced a green and one-pot method for the fabrication of bright Cdots, Fig. 1a. The Cdots was synthesized based on the hydrothermal carbonization of freshly synthesized BSA nanoparticles. Firstly, BSA proteins were denatured in ethanol solution for 5 min. This treatment effectively breaks the native three-dimensional structure of BSA and makes the carboxyl and amine groups be extensively exposed. By adding few amount of glutaraldehyde (8%, 25 μL) into the solution and keeping the solution stirred for 12 h, the denatured BSA proteins can be cross-linked together and self-assembled into a compact spherical structure with size around 40 nm (Fig. S1). The resultant BSA nanoparticles were then transferred into DI water and put in a poly (tetrafluoroethylene) (Teflon)-lined autoclave and heated at different conditions as noted below.

### Spectroscopic and Microscopic Characterizations

Fig. 1b shows the typical fluorescence spectra of BSA Cdots. At 150 °C, the carbonization process is very slow. In comparison with the fluorescence spectra of BSA nanoparticles before the carbonization process, the fluorescence intensity of the resultant nanoparticles was decreased gradually as a function of time (reduced around 60% after 8 h). However, the maximum of emission (525 nm) and excitation wavelength (480 nm) were still staying in the same location even reacted for 8 h, indicative of a partial carbonization process, Fig. S2. When the reaction temperature was increased to 200 °C, tens of nanometer blue shifts were observed immediately in both of the excitation (from 480 to 380 nm) and emission spectra (from 525 to 450 nm) after 2 h. The color of the solution was changed from pale brown into deep brown. Further extending the reaction time, the fluorescence intensity of the resultant product reached a maximum at 6 h, Fig. S3. Interestingly, the emission wavelength of these Cdots can be readily adjusted by modulating the excitation wavelength. When the excitation light was shifted to longer wavelength, the maximum of emission was changed to the same direction simultaneously, Fig. 1c. Analogous feature was also observed from the Cdots made by other precursors, which would be mainly ascribed to the heterogeneous surface defects as well as inhomogeneous size distribution^16^,^19^,^42^. This property should be of great interest for fluorescence based imaging at cellular environment because longer excitation wavelength enables the differentiation of background noise more feasible.

**Figure 2:**
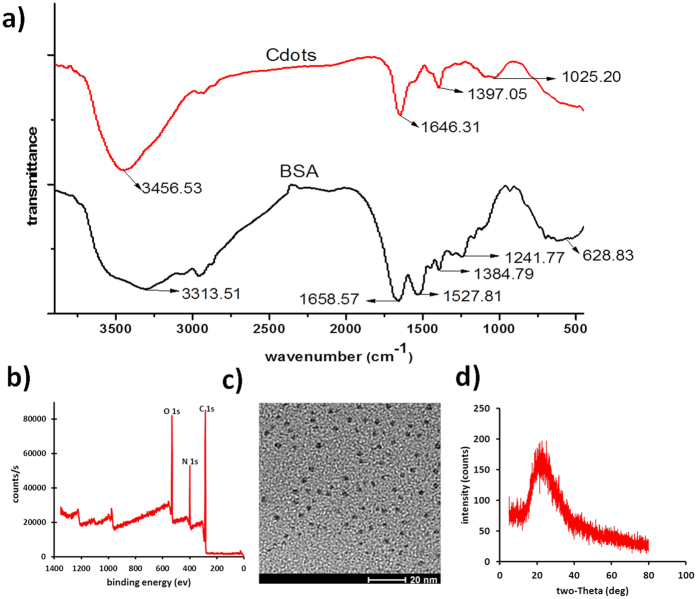
(**a**) FT-IR spectra of BSA nanoparticles (black) and Cdots (red). (**b**) XPS spectrum, (**c**) TEM image and d) XRD spectrum of Cdots.

In order to confirm the carbonization process, FT-IR spectroscopy was applied to characterize the variation of the chemical structure of BSA nanoparticles, Fig. 2a. Initially, the BSA nanoparticle exhibits complex fingerprint peaks owing to the distinctive chemical structure of amino acids. After the carbonization process, the spectrum becomes simpler and parts of peaks slightly shift because of the changed chemical environment. In the FT-IR spectrum of Cdots, several representative peaks can still be found such as the peak at 3456 cm^−1^ corresponds to the typical –OH stretching mode, the peaks at about 1646, 1025 cm^−1^ are due to –COO^-^, implying the existence of abundant of carboxyl groups. According to these results, it can envision that the application capability of these Cdots could be further extended to bioimaging, medical diagnosis as well as drug delivery because no further chemical treatment was required for the surface modification. In addition, the abundance of –OH and –COOH groups covalently bonded on the framework can greatly improve the hydrophilicity and stability of the nanoparticles in aqueous solution. Elemental analysis shows that the Cdots are composed of C 70 wt%, N 14 wt% and O 16 wt%, Fig. 2b.

In a parallel experiment, we carbonized BSA proteins directly (without the cross-linking process) under the optimum reaction conditions. As shown in Fig. S4, the fluorescence intensity of the resultant nanoparticles is almost 4 × weaker than that made by cross-linked BSA nanoparticles. This observation is well consistent with the previous observation that an initial polymerization process is normally involved in for the successful generation of highly luminescent Cdots^27^. By selecting quinine sulfate as the standard and 380 nm as the excitation wavelength, the photoluminescence Q.Y. of the Cdots made by this compact BSA nanoparticles was measured and calculated to be 38.4%. To the best of our knowledge, our result is better than the previous work that also generated from proteins^43^,^44^,^45^.

To gain morphology information, TEM image of the Cdots was obtained. As revealed in Fig. 2c, the Cdots are evenly dispersed on the substrate without noticeable aggregation and display a uniform size distribution (with diameter around 2.2 ± 0.4 nm). This observation is well in agreement with the FT-IR analysis that the –OH and –COOH indeed effectively stabilize the Cdots in water solution. The absence of discernible lattice structures on the high resolution TEM image indicates that the resultant Cdots are amorphous. This conclusion is also consistent with X-ray diffraction measurements. As demonstrated in Fig. 2d, a broad peak located at 2θ = 25^o^, suggesting an amorphous nature.

### Colloidal and fluorescence stability estimations

From the consideration of their potential chemical and biological application capability, it is significant to explore the stability of these carbon nanoparticles not only from the aspect of colloidal stability but also from the performance of the optical property resistant to chemical stimuli.

**Figure 3:**
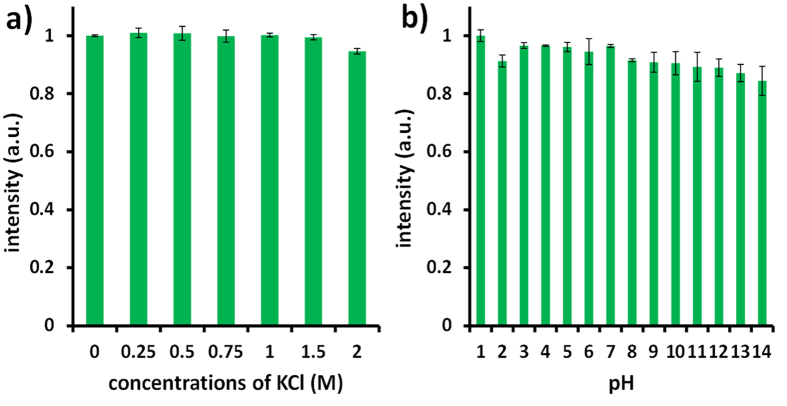
The effect of salt (**a**) and pH (**b**) on the fluorescence intensity of Cdots.

For the first aspect, since most of the biological or chemical samples typically exist in buffer solutions containing sodium or potassium, the dispersiveness of the Cdots in salt solutions with different concentrations should be explored in advance. As shown in Fig. 3a, the fluorescence intensity of Cdots in a set of KCl solutions with different concentrations was measured. Even in the solution with salt concentration higher than 150 mM, the fluorescence intensity of Cdots is still comparable with that in DI water, indicative of the superior colloidal stability against the particle/particle attraction force, e.g. van der Waals interactions. The negligible fluorescence fluctuation over a broad range of salt concentrations demonstrates that the Cdots are suitable for biological imaging applications. In comparison with other well-studied fluorescent nanomaterials such as semiconducting quantum dots, additional and complicated ligand exchange processes are typically required for the generation of good water solubility and colloidal stability^4^,^6^.

To realize fluorescence-based single particle imaging or biosensing assay, one of the essential prerequisites is to make sure that the probe is stable in complicated chemical solutions. On this basis, the Cdots was treated in a set of solutions with different pH values for 2 h and then compared the fluorescence intensity with that in DI water. As shown in Fig. 3b, the as-synthesized Cdots exhibit decent fluorescence stability from pH 1 to 14. In contrast to the Cdots made by other methods^46^,^47^, the Cdots synthesized by this method is insensitive to the pH change of the surrounding. Besides the factor of pH, we further explored the influence of other chemicals, i.e. H_2_O_2_ and NaClO, which can irreversibly influence the fluorescence of many dyes and nanomaterials. Fig. S5 shows the fluorescence intensity fluctuations of Cdots in a set of H_2_O_2_ and NaClO solutions. Interestingly, no noticeable fluorescence quenching effect was observed even when the concentration of H_2_O_2_ and NaClO is as high as 300 μM. This feature might be benefit from the inner chemical structure of carbonized material as well as the characteristic photoluminescence mechanism of this quantum-sized nanodots. The reactive chemical groups are not able to influence the conversion process of electron-hole pairs in Cdots.

### Metal ion sensing

**Figure 4:**
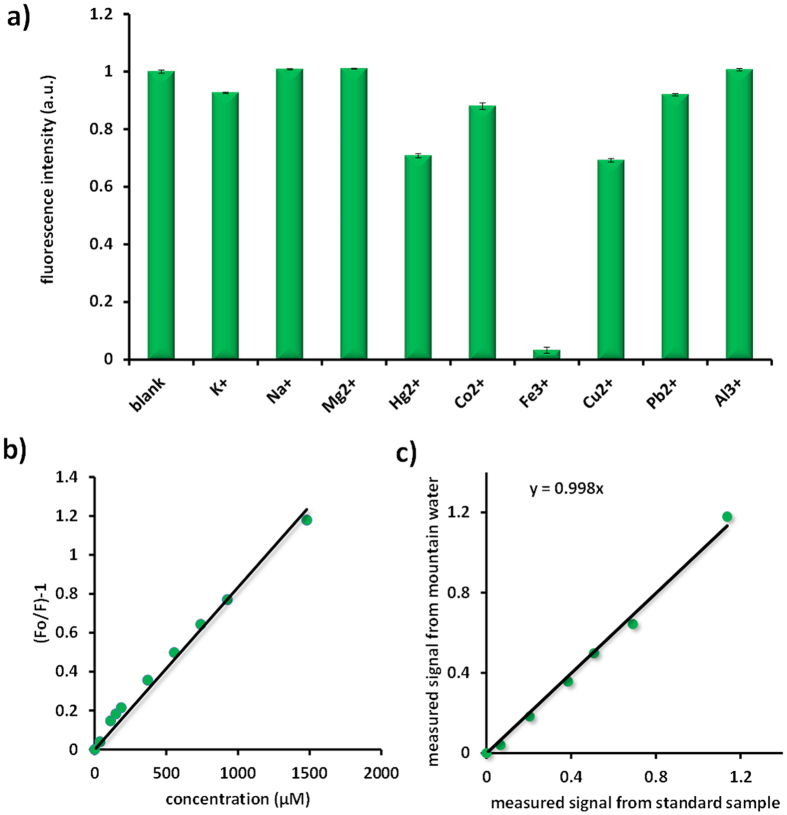
(**a**) The metal ion dependent fluorescence quenching effect of Cdots in different metal ion solutions with [M^n+^] = 2 mM. (**b**) The calibration curve of Cdots as a function of Fe^3+^ concentration in the solution. (**c**) The recovery curve of mountain spring water spiked with Fe^3+^.

Because of the vital roles of metal ions in biological and environmental applications, great efforts have been devoted to develop convenient yet sensitive detection schemes for the ultrasensitive detection of metal ions at the micromolar level^17^,^45^,^48^,^49^,^50^. As confirmed by FT-IR spectrum, the surface of as-synthesized Cdots was encapsulated with many carboxyl and hydroxyl groups. They would therefore provide a versatile route for the coordination of specific cationic metal ions, which could quench the fluorescence of the CDs owing to the nonradiative electron-transfer that involves partial transfer of an electron in the excited state to the d orbital of Fe^3+^^27^. Indeed, the Cdots display a high selectivity toward Fe^3+^ among other metal ions, including K^+^, Na^+^, Mg^2+^, Hg^2+^, Co^2+^, Cu^2+^, Pb^2+^ and Al^3+^, Fig. 4a.

It is worth to note that Fe^3+^ plays significant roles in many biochemical processes, such as transport of oxygen to tissues. The disorders of Fe^3+^ in its metabolism will result in anemia, liver and kidney damage, diabetes, and heart failure^51^,^52^. Monitoring the concentration of Fe^3+^ in the drink water is thus fundamentally important. Fig. 4b shows the fluorescence quenching curve of Cdots at the wavelength of 450 nm as a function of Fe^3+^ concentration (from 0 μM to 1.5 mM). The quenching curve can be fitted by the Stern-Volmer equation F_o_/F-1 = K_sv_C well, where K_sv_ is the Stern-Volmer quenching constant, C is the analyte concentration, and F_0_ and F are the fluorescence intensities of Cdots at 450 nm in the absence and presence of Fe^3+^ respectively. The detection limit is estimated to be 0.5 μM, which is comparable to the previously reported results by using Cdots as the probe^48^,^49^. In addition, the linear dynamic range of our probe for Fe^3+^ sensing is much broader over the previously reported results measured by Cdots and comparable with the results achieved by fluorescent gold cluster^53^.

In order to evaluate the performance of this sensor in real sample analysis, as a proof of concept experiment, we used the water from the springs at Yuelu Mountain (Hunan Province, China, Many climbers drank the water directly) and spiked with Fe^3+^ at different concentrations. The fluorescence intensity of the Cdots decreased gradually when the concentration of Fe^3+^ was increased (from 37 μM to 1.5 mM). The plot of the measured Fe^3+^ as a function of the content spiked is shown in Fig. 4c. Although many trace metal ions might exist in the river water, the good linear relationship (with a slope of 0.998) of this recovery cure indicates that the Cdots can specifically and accurately distinguish Fe^3+^ in the spring water. It is worth to note that the interferences from phosphates, chlorides and carbonates which might exist in the spring water are negligible, Fig. S6. These results confirm that this Fe^3+^ sensor is potentially useful for real sample analysis in the future.

### Single particle imaging and dynamic tracking on living cell membrane

**Figure 5:**
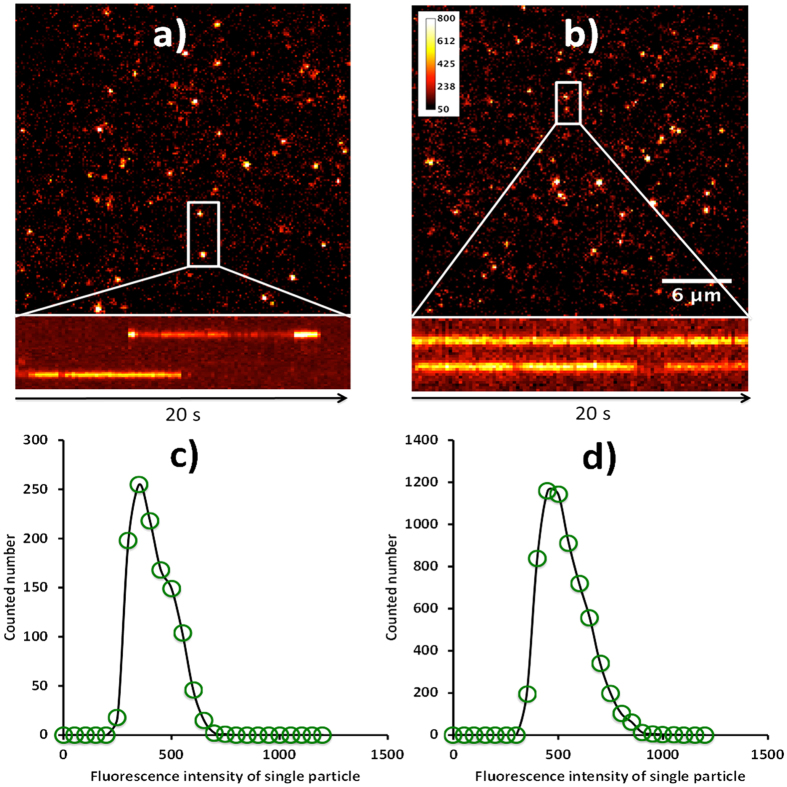
The fluorescence microscopic images of quantum dots (**a**) and Cdots (**b**) with excitation at 532 nm. (**c,d**) are the corresponding single particle intensity distribution histograms of quantum dots and Cdots respectively.

Besides the metal ion sensing applications, it is also interesting to explore the fluorescence imaging capability of the Cdots in solution or in biological samples at single particle level. In this regard, we studied the fluorescence properties of Cdots at single particle level with an optical microscope. In order to continuously image individual Cdots in solution, we modified the glass slide with APTMS. The electrostatic interaction enables the Cdots being stably adsorbed on the glass slide surface. The unbounded Cdots in the solution were then washed away. As shown in Fig. 5, the Cdots display bright fluorescence and are dispersed separately on the glass slide surface. The fluorescence intensity of the Cdots is evenly distributed, Fig. 5d, which is well in agreement with the size distribution measurements. For fluorescence-based single molecule/particle imaging applications, there are two essential parameters required to be understand in advance, i.e. photobleaching and photoblinking effects.

Here, we chose the most commonly adopted fluorescent contrast reagent semiconducting quantum dots (Qdots, with maximum emission at 530 nm) as a control. A typical fluorescence image of single Qdots on the glass slide surface is shown in Fig. 5a. The detected fluorescence intensity of individual Qdots (with mean gray value of 400 in the fluorescence image after subtracted the background noise) is comparable with that from single Cdots (with mean gray value of 450) with the excitation wavelength at 532 nm and exposure time of 10 ms. From the time dependent fluorescence tracks, the majority of Qdots exhibit inherent frequent transitions between on and off states (more than 95% from 80 points). However, this drawback is greatly improved in the case of Cdots as indicated in the representative tracks, Fig. 5a,b. More importantly, no evident photo-induced bleaching was observed from both of Qdots and Cdots under this imaging setup (the laser power is 1 mW).

The superior optical stability of Cdots against photoblinking and photobleaching indicates that they are promising fluorescent imaging contrast reagent for dynamic events tracking at single object level. In this regard, we further conjugated the Cdots with cell penetration peptide (CPP) and explored the capability of these Cdots for dynamic tracking on living cell membrane. As a proof of concept experiment, we chose the transacting activator of transcription (TAT) peptide (residues 47–57: YGRKKRRQRRR) derived from human immunodeficiency virus (HIV-1) as a model system and then conjugated with Cdots through the linkers of EDC/NHS^54^,^55^. After covalently conjugation of this cationic peptide on the surface of nanocargo, a broad variety of functional materials including various nanoparticulate pharmaceutical carriers (liposomes, micelles, nanoparticles) have been successfully introduced into living cells with high efficiency^55^,^56^.

**Figure 6:**
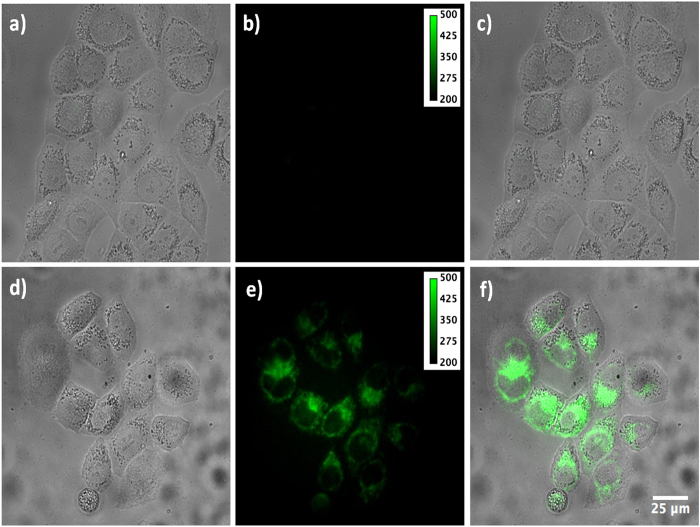
Cellular labeling experiments. From (**a**–**c**) are the bright field, fluorescence and overlapped images of HeLa cells treated with Cdots without TAT peptides conjugation, respectively. From (**d**–**f**) are the bright field, fluorescence and overlapped images of HeLa cells treated with TAT peptides modified with Cdots, respectively.

Fig. 6 shows the cellular uptake results of TAT-modified Cdots. After co-incubation of TAT-Cdots with HeLa cell for 2 h at 37 °C, bright fluorescence from Cdots can be readily observed inside the cytosol. Interestingly, in the control experiment where Cdots without TAT modification, no detectable fluorescence was found under the same incubation and illumination conditions, indicative of negligible nonspecific interactions with living cell membrane. The feature of avoiding nonspecific interactions with cell membrane is significantly important in diverse areas, especially for cell diagnosis and target drug delivery in nanomedicine. Therefore, the Cdots prepared at here would be a promising candidate for specific cellular imaging as well as gene and drug delivery in living cell system.

**Figure 7:**
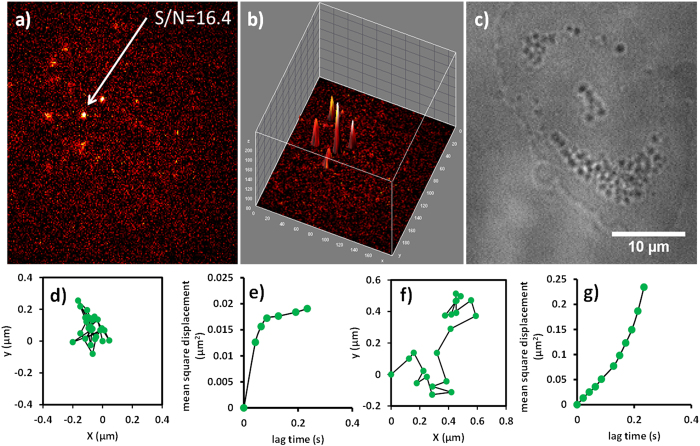
Single particle tracking experiments of TAT peptides modified Cdots on living cell membrane. (**a**) The fluorescence image of Cdots on living cell membrane. (**b**) The corresponding three dimensional intensity profile image of **(a)**. (**c**) The bright field image of the observed cell. (**d,f**) are two typical single particle diffusion trajectories of Cdots on living cell membrane with diffusion coefficient of (0.06 and 0.15 μm^2^/s) respectively. (**e,g**) are the corresponding mean square displacement (MSD) plot as a function of lag time.

We then further studied the single particle tracking capability of these TAT-Cdots on living cell membrane. To selectively image TAT-Cdots on living cell membrane, we used a highly inclined thin illumination mode to excite the fluorescence from Cdots. The particles out of the focal plane (the cell membrane) will not be observed. In addition, for a particle with size around 10 nm, the diffusion coefficient is around 21 μm^2^/s in water. On this account, it is a grand challenge to track the TAT-Cdots in free solution with a temporal resolution of 47 Hz. Fig. 7 shows the representative fluorescence image of individual TAT-Cdots on living HeLa cell membrane. For single molecule fluorescence imaging in living cell system, parts of proteins in the cytosol can result in undesired interfering fluorescence, making the target object hardly distinguishable. As indicated in Fig. 7a, when the Cdots is locating at the focal plane of the objective, the TAT-Cdots still exhibited signal to noise as high as 16, which is good enough for high precision single particle tracking on living cell membrane^56^,^57^,^58^,^59^.

Previous experimental results have demonstrated that the thermal energy induced Brownian diffusion of TAT-modified nanoparticles on lipid bilayer can be greatly inhibited owing to the multi-interaction points such as hydrogen bonds^59^. On living cell membrane, the translational diffusion of TAT-modified nanocargo exhibited confined behavior even in the presence of many neutral sugar molecules on the cell surface^56^. Although the size dimension of Cdots is far smaller than the nanoparticles used before, in this experiment, we found that the majority of TAT-Cdots still display analogous restricted diffusion once they bound to the cell membrane as indicated in Fig. 7d. Interestingly, a distinctive phenomenon noted here is that the binding strength of the Cdots with the cell membrane is not as strong as that from TAT-modified 60 nm gold nanoparticles. Parts of the Cdots could diffuse away after they bound on the cell membrane. And few of them even show directed diffusion on the cell membrane, Fig. 7f. This might be owing to the much smaller size dimension of Cdots where fewer TAT peptides could be anchored on the Cdots surface, resulting weaker binding strength and higher movability^60^.

In summary, we demonstrated a convenient and green strategy for the one-pot synthesis of water soluble and bright Cdots. The as-synthesized Cdots exhibit narrow size distribution and good colloidal stability in different surroundings such as concentrated salt solutions, acid and basic environments. The abundance of carboxyl and hydroxyl groups on the Cdots surface not only play a central role for the effective stabilization of Cdots in different surroundings but also afford a versatile platform for the specific recognition of metal ions, i.e. Fe^3+^ in spring water. Furthermore, the single particle fluorescence characterizations illustrate that the Cdots exhibit superior optical features such as improved photoblinking and good photostability comparable to semiconducting Qdots under the same illumination conditions, which is significant for cellular imaging and single particle tracking applications. In contrast to the previously reported strategies for Cdots fabrication, the method demonstrated herein doesn’t require post surface modification for the improvement of fluorescence brightness as well as water stability. We believed that the Cdots prepared by this strategy would display promising applications in various areas, including analytical chemistry, nanomedicine, biochemistry and so on.

## Methods

### Chemicals and Materials

Bovine serum albumin, ethanol, glutaraldehyde, 1-ethyl-3-(-3-dimethylaminopropyl) carbodiimide hydrochloride (EDC) and *N*-hydroxysulfosuccinimide (NHS) were obtained from Sigma-Aldrich (St. Louis, MO, U.S.A.). NH_2_-TAT peptide (sequence, YGRKKRRQRRR) was purchased from AnaSpec. (San Jose, CA, U.S.A.). All other chemicals not mentioned here were purchased from Sigma-Aldrich.

### BSA Nanoparticles and BSA Cdots Fabrication and Characterization

The fluorescent BSA Cdots were synthesized by carbonization of freshly synthesized BSA nanoparticles in deionized (DI) water. To prepare BSA nanoparticles, 20 mg of BSA powder was dissolved in 1 mL DI water and then 2 mL ethanol was gradually added into the solution and kept stirred for 5 min. After the denaturation process, 25 μL of 8% glutaraldehyde was added to the mixture and kept stirred for 18 h. The resulted BSA nanoparticles were washed with DI water for three times by centrifugal ultrafiltration (100 K).

To synthesize fluorescent BSA Cdots, typically, 300 μL of freshly prepared BSA nanoparticles was transferred into a 10 mL Teflon-linked stainless steel autoclave and heated at 200 °C for 6 h and then cooled to room temperature naturally followed with sonication. The aqueous solution was subsequently washed with centrifugal ultrafiltration (100 K) three times. The brown aqueous solution containing BSA Cdots was stored at 4 °C before usage.

The size of BSA Cdots was characterized by transmission electron microscopy (TEM) (JEM 1230, JEOL, Japan). The sample for TEM analysis was transferred into ethanol solution first and then dropt 2 μL of the product onto carbon coated copper grids. X-ray Photoelectron Spectroscopy (XPS) was investigated by using K-Alpha 1063 (Thermo Fisher Scientific) with a mono X-ray source Al Ka excitation. Binding energy calibration was based on C1s at 284.6 eV. Elemental analysis was performed on Thermo Avantage, each data was parallel at least twice and the average values were obtained by measuring three kinds of sample batches. The X-ray Diffraction (XRD) analysis was performed using D/max-2500 (Rigaku, Japan). The UV-vis absorption spectrum was measured by Specord S600 (Analytik Jena, Germany). Fluorescence spectroscopy analysis was conducted on a Hitachi F-7000 spectrophotometer (Hitachi, Ltd., Japan). FT-IR spectra were measured with a Thermo Nicolet 6700 spectrometer (Thermo Fisher Scientific Inc. U.S.A.) ranging from 650 to 4000 cm^−1^. The fluorescence Q.Y. of BSA Cdots was measured by using quinine sulfate (Q.Y. 54%) as a reference. The quinine sulfate was dissolved in 0.1 M H_2_SO_4_. By using the equation 
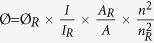
 , where 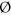 is the measured Q.Y. of Cdots, 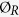 is the Q.Y. of quinine sulfate, *I* is integrated fluorescence intensity, *n* and *A* are the refractive index and optical density of the solution, the relative Q.Y. of Cdots can then be readily determined.

### Conjugation of TAT Peptides with BSA Cdots

Conjugation of TAT peptides with Cdots is based on EDC/NHS crosslinking reaction^56^,^59^. Briefly, 0.25 mL of purified Cdots solution was mixed with 5 μL of 1 mg/mL EDC and 12 μL of 1 mg/mL NHS for 30 min. TAT (5 μL, 0.5 mg/mL) was then added to the mixture and kept stirred overnight. After the conjugation process, additional unreacted reagents were washed away by centrifugal ultrafiltration (100 K). The purified TAT-Cdots were suspended in deionized water and stored at 4 °C prior to usage.

### Cell Culture

Cervical cancer HeLa cells were obtained from American Type Culture Collection (ATCC, U.S.A). HeLa cells were cultured on a cleaned glass slide in a plastic cell culture dish. Before the cellular uptake and single particle imaging studies, the cells were maintained in Dulbecco’s modified Eagle’s medium (DMEM, Gibco, Thermo Fisher Scientific Inc.) supplemented with 10% fatal bovine serum (Gibco, Thermo Fisher Scientific Inc.) at 37 °C/5% CO_2_ in a humidified atmosphere.

### Imaging of individual BSA Cdots on Glass Slide and Living Cell Membrane

Single particle fluorescence imaging experiments were performed on an inverted optical microscope (Ti-U, Nikon, Japan). The mercury lamp was replaced with a solid-state diode laser (532 nm, CNI laser Changchun, China). The expended laser line was reflected with a dichroic mirror and focused onto the back port of an oil immersion objective (NA 1.49, 100 × , Nikon, Japan). The emitted fluorescence from the sample was then filtered with a band pass filter (605/55, Semrock, U.S.A.) In order to reduce the background noise from the sample and to image only the cell membrane, the incidence angle of the laser line was slightly adjusted to achieve a highly inclined thin illumination mode. The fluorescence image was captured by an EMCCD camera (Ultra 897, Andor, UK). The exposure time was set to 20 ms. Under this condition the data acquisition rate can reach 47 Hz for dynamic tracking.

The diffusion trajectories of TAT-Cdots on cell membrane were analyzed with Image J (http://rsb.info.nih.gov/ij/) and Matlab. The detailed procedures for single particle trajectory analysis can be found in the previous work^57^. With this imaging setup (the pixel size of the camera is 16 μm × 16 μm, that is in the image plane each pixel represents an area of 160 nm×160 nm), the temporal localization accuracy (with S/N better than 10) is comparable to the results in our previous work (better than 10 nm)^56^,^58^,^59^.

## Additional Information

**How to cite this article**: Yang, Q. *et al.* Single Particle Dynamic Imaging and Fe^3+^ Sensing with Bright Carbon Dots Derived from Bovine Serum Albumin Proteins. *Sci. Rep.* **5**, 17727; doi: 10.1038/srep17727 (2015).

## Supplementary Material

Supplementary Information

## References

[b1] Ozawa T., Yoshimura H. & Kim S. B. Advances in fluorescence and bioluminescence imaging. Anal. Chem. 85, 590–609 (2013). 23134415 10.1021/ac3031724

[b2] Jain P. K., Lee K. S., El-Sayed I. H. & El-Sayed M. A. Calculated absorption and scattering properties of gold nanoparticles of different size, shape, and composition: applications in biological imaging and biomedicine. J. Phys. Chem. B 110, 7238–7248 (2006). 16599493 10.1021/jp057170o

[b3] Xiao L. & Yeung E. S. Optical imaging of individual plasmonic nanoparticles in biological samples. Annu Rev Anal Chem 7, 89–111 (2014). 10.1146/annurev-anchem-071213-020125 24818812

[b4] Chan W. C. & Nie S. Quantum dot bioconjugates for ultrasensitive nonisotopic detection. Science 281, 2016–2018 (1998). 9748158 10.1126/science.281.5385.2016

[b5] Bruchez M., Moronne M., Gin P., Weiss S. & Alivisatos A. P. Semiconductor nanocrystals as fluorescent biological labels. Science 281, 2013–2016 (1998). 9748157 10.1126/science.281.5385.2013

[b6] Alivisatos A. P., Gu W. & Larabell C. Quantum dots as cellular probes. Annu. Rev. Biomed. Eng. 7, 55–76 (2005). 16004566 10.1146/annurev.bioeng.7.060804.100432

[b7] Nie S., Xing Y., Kim G. J. & Simons J. W. Nanotechnology applications in cancer. Annu. Rev. Biomed. Eng. 9, 257–288 (2007). 17439359 10.1146/annurev.bioeng.9.060906.152025

[b8] Resch-Genger U., Grabolle M., Cavaliere-Jaricot S., Nitschke R. & Nann T. Quantum dots versus organic dyes as fluorescent labels. Nat. Meth. 5, 763–775 (2008). 10.1038/nmeth.1248 18756197

[b9] Chan W.-H. & Shiao N.-H. Cytotoxic effect of CdSe quantum dots on mouse embryonic development. Acta Pharmacol. Sin. 29, 259–266 (2008). 18215357 10.1111/j.1745-7254.2008.00743.x

[b10] Kirchner C. *et al.* Cytotoxicity of colloidal CdSe and CdSeZnS nanoparticles. Nano Lett. 5, 331–338 (2005). 15794621 10.1021/nl047996m

[b11] Derfus A. M., Chan W. C. W. & Bhatia S. N. Probing the cytotoxicity of semiconductor quantum dots. Nano Lett. 4, 11–18 (2004). 10.1021/nl0347334 PMC5588688 28890669

[b12] Hoshino A. *et al.* Physicochemical properties and cellular toxicity of nanocrystal quantum dots depend on their surface modification. Nano Lett. 4, 2163–2169 (2004).

[b13] Zheng J., Nicovich P. R. & Dickson R. M. Highly fluorescent noble-metal quantum dots. Annu. Rev. Phys. Chem. 58, 409–431 (2007). 17105412 10.1146/annurev.physchem.58.032806.104546 PMC2735021

[b14] He Y. *et al.* Ultrastable, highly fluorescent, and water-dispersed silicon-based nanospheres as cellular probes. Angew. Chem. Int. Ed. 48, 128–132 (2008). 10.1002/anie.200802230 18979474

[b15] Xu X. *et al.* Electrophoretic analysis and purification of fluorescent single-walled carbon nanotube fragments. J. Am. Chem. Soc. 126, 12736–12737 (2004). 15469243 10.1021/ja040082h

[b16] Sun Y.-P. *et al.* Quantum-sized carbon dots for bright and colorful photoluminescence. J. Am. Chem. Soc. 128, 7756–7757 (2006). 16771487 10.1021/ja062677d

[b17] Liu X., Zhang N., Bing T. & Shangguan D. Carbon dots based dual-emission silica nanoparticles as a ratiometric nanosensor for Cu^2+^. Anal. Chem. 86, 2289–2296 (2014). 24476566 10.1021/ac404236y

[b18] Cao L. *et al.* Carbon dots for multiphoton bioimaging. J. Am. Chem. Soc. 129, 11318–11319 (2007). 17722926 10.1021/ja073527l PMC2691414

[b19] Liu H., Ye T. & Mao C. Fluorescent carbon nanoparticles derived from candle soot. Angew. Chem. Int. Ed. 46, 6473–6475 (2007). 10.1002/anie.200701271 17645271

[b20] Barone P. W., Baik S., Heller D. A. & Strano M. S. Near-infrared optical sensors based on single-walled carbon nanotubes. Nat. Mater. 4, 86–92 (2005). 15592477 10.1038/nmat1276

[b21] Welsher K. *et al.* A route to brightly fluorescent carbon nanotubes for near-infrared imaging in mice. Nat. Nanotech. 4, 773–780 (2009). 10.1038/nnano.2009.294 PMC2834239 19893526

[b22] Cao L., Meziani M. J., Sahu S. & Sun Y.-P. Photoluminescence properties of graphene versus other carbon nanomaterials. Acc. Chem. Res. 46, 171–180 (2013). 23092181 10.1021/ar300128j

[b23] Wei L. *et al.* Fabrication of bright and small size semiconducting polymer nanoparticles for cellular labelling and single particle tracking. Nanoscale 6, 11351–11358 (2014). 25141182 10.1039/c4nr03293d

[b24] Li H. *et al.* Water-soluble fluorescent carbon quantum dots and photocatalyst design. Angew. Chem. Int. Ed. 49, 4430–4434 (2010). 10.1002/anie.200906154 20461744

[b25] Zhu W., Zhang J., Jiang Z., Wang W. & Liu X. High-quality carbon dots: synthesis, peroxidase-like activity and their application in the detection of H_2_O_2_, Ag^+^ and Fe^3+^. RSC Adv. 4, 17387–17392 (2014).

[b26] Ding C., Zhu A. & Tian Y. Functional surface engineering of C-Dots for fluorescent biosensing and *in vivo* bioimaging. Acc. Chem. Res. 47, 20–30 (2014). 23911118 10.1021/ar400023s

[b27] Zhu S. *et al.* Highly Photoluminescent carbon dots for multicolor patterning, sensors, and bioimaging. Angew. Chem. Int. Ed. 52, 3953–3957 (2013). 10.1002/anie.201300519 23450679

[b28] DuChene J. S. *et al.* Halide anions as shape-directing agents for obtaining high-quality anisotropic gold nanostructures. Chem. Mater. 25, 1392–1399 (2013).

[b29] Hola K. *et al.* Carbon dots-emerging light emitters for bioimaging, cancer therapy and optoelectronics. Nano Today 9, 590–603 (2014).

[b30] Lim S. Y., Shen W. & Gao Z. Carbon quantum dots and their applications. Chem. Soc. Rev. 44, 362–381 (2014). 25316556 10.1039/c4cs00269e

[b31] Guo X., Wang C.-F., Yu Z.-Y., Chen L. & Chen S. Facile access to versatile fluorescent carbon dots toward light-emitting diodes. Chem. Commun. 48, 2692–2694 (2012). 10.1039/c2cc17769b 22306963

[b32] Ge J. *et al.* Red‐emissive carbon dots for fluorescent, photoacoustic, and thermal theranostics in living mice. Adv. Mater. 27, 4169–4177 (2015). 26045099 10.1002/adma.201500323

[b33] Ge J. *et al.* A graphene quantum dot photodynamic therapy agent with high singlet oxygen generation. Nat. Commun. 5, 4596 (2014). 25105845 10.1038/ncomms5596 PMC4143951

[b34] Lu J. *et al.* One-pot synthesis of fluorescent carbon nanoribbons, nanoparticles, and graphene by the exfoliation of graphite in ionic liquids. ACS Nano 3, 2367–2375 (2009). 19702326 10.1021/nn900546b

[b35] Zheng L., Chi Y., Dong Y., Lin J. & Wang B. Electrochemiluminescence of water-soluble carbon nanocrystals released electrochemically from graphite. J. Am. Chem. Soc. 131, 4564–4565 (2009). 19296587 10.1021/ja809073f

[b36] Li Y. *et al.* An Electrochemical avenue to green-luminescent graphene quantum dots as potential electron-acceptors for photovoltaics. Adv. Mater. 23, 776–780 (2010). 21287641 10.1002/adma.201003819

[b37] Zhu H. *et al.* Microwave synthesis of fluorescent carbon nanoparticles with electrochemiluminescence properties. Chem. Commun. 34, 5118–5120 (2009). 10.1039/b907612c 20448965

[b38] Mochalin V. N. & Gogotsi Y. Wet chemistry route to hydrophobic blue fluorescent nanodiamond. J. Am. Chem. Soc. 131, 4594–4595 (2009). 19290627 10.1021/ja9004514

[b39] Qiao Z.-A. *et al.* Commercially activated carbon as the source for producing multicolor photoluminescent carbon dots by chemical oxidation. Chem. Commun. 46, 8812–8814 (2010). 10.1039/c0cc02724c 20953494

[b40] Chen P.-C., Chen Y.-N., Hsu P.-C., Shih C.-C. & Chang H.-T. Photoluminescent organosilane-functionalized carbon dots as temperature probes. Chem. Commun. 49, 1639–1641 (2013). 10.1039/c3cc38486a 23340928

[b41] Hsu P.-C. & Chang H.-T. Synthesis of high-quality carbon nanodots from hydrophilic compounds: role of functional groups. Chem. Commun. 48, 3984–3986 (2012). 10.1039/c2cc30188a 22422194

[b42] Liu R. *et al.* An aqueous route to multicolor photoluminescent carbon dots using silica spheres as carriers. Angew. Chem. Int. Ed. 48, 4598–4601 (2009). 10.1002/anie.200900652 19388019

[b43] Zhang Z., Hao J., Zhang J., Zhang B. & Tang J. Protein as the source for synthesizing fluorescent carbon dots by a one-pot hydrothermal route. RSC Adv. 2, 8599–8601 (2012).

[b44] Wang L. & Zhou H. S. Green synthesis of luminescent nitrogen-doped carbon dots from milk and its imaging application. Anal. Chem. 86, 8902–8905 (2014). 25181643 10.1021/ac502646x

[b45] Wee S. S., Ng Y. H. & Ng S. M. Synthesis of fluorescent carbon dots via simple acid hydrolysis of bovine serum albumin and its potential as sensitive sensing probe for lead (II) ions. Talanta 116, 71–76 (2013). 24148375 10.1016/j.talanta.2013.04.081

[b46] Qu S., Chen H., Zheng X., Cao J. & Liu X. Ratiometric fluorescent nanosensor based on water soluble carbon nanodots with multiple sensing capacities. Nanoscale 5, 5514–5518 (2013). 23673389 10.1039/c3nr00619k

[b47] Jia X., Li J. & Wang E. One-pot green synthesis of optically pH-sensitive carbon dots with upconversion luminescence. Nanoscale 4, 5572–5575 (2012). 22786671 10.1039/c2nr31319g

[b48] Xu J., Zhou Y., Liu S., Dong M. & Huang C. Low-cost synthesis of carbon nanodots from natural products used as a fluorescent probe for the detection of ferrum(III) ions in lake water. Anal. Methods 6, 2086–2090 (2014).

[b49] Zhou L., Geng J. & Liu B. Graphene quantum dots from polycyclic aromatic hydrocarbon for bioimaging and sensing of Fe^3+^ and hydrogen peroxide. Part. Part. Syst. Charact. 30, 1086–1092 (2013).

[b50] Lu W. *et al.* Economical, green synthesis of fluorescent carbon nanoparticles and their use as probes for sensitive and selective detection of mercury(II) ions. Anal. Chem. 84, 5351–5357 (2012). 22681704 10.1021/ac3007939

[b51] Brugnara C. Iron deficiency and erythropoiesis: new diagnostic approaches. Clin. Chem. 49, 1573–1578 (2003). 14500582 10.1373/49.10.1573

[b52] Cheney K., Gumbiner C., Benson B. & Tenenbein M. Survival after a severe iron poisoning treated with intermittent infusions of deferoxamine. Clin. Toxicol. 33, 61–66 (1995). 10.3109/15563659509020217 7837315

[b53] Annie Ho, J.-A., Chang H.-C. & Su W.-T. DOPA-mediated reduction allows the facile synthesis of fluorescent gold nanoclusters for use as sensing probes for ferric ions. Anal. Chem. 84, 3246–3253 (2012). 22364482 10.1021/ac203362g

[b54] Zorko M. & Langel U. Cell-penetrating peptides: mechanism and kinetics of cargo delivery. Adv. Drug Deliv. Rev. 57, 529–545 (2005). 15722162 10.1016/j.addr.2004.10.010

[b55] Brooks H., Lebleu B. & Vives E. Tat peptide-mediated cellular delivery: back to basics. Adv. Drug Deliv. Rev. 57, 559–577 (2005). 15722164 10.1016/j.addr.2004.12.001

[b56] Wei L., Yang Q. & Xiao L. Tempo-spatially resolved cellular dynamics of human immunodeficiency virus transacting activator of transcription (Tat) peptide-modified nanocargos in living cells. Nanoscale 6, 10207–10215 (2014). 25051531 10.1039/c4nr02732a

[b57] Sbalzarini I. F. & Koumoutsakos P. Feature point tracking and trajectory analysis for video imaging in cell biology. J. Struct. Biol. 151, 182–195 (2005). 16043363 10.1016/j.jsb.2005.06.002

[b58] Xiao L., Wei L., Liu C., He Y. & Yeung E. S. Unsynchronized translational and rotational diffusion of nanocargo on a living cell membrane. Angew. Chem. Int. Ed. 51, 4181–4184 (2012). 10.1002/anie.201108647 22431379

[b59] Wei L. *et al.* Frozen Translational and rotational motion of human immunodeficiency virus transacting activator of transcription peptide-modified nanocargo on neutral lipid bilayer. Anal. Chem. 85, 5169–5175 (2013). 23581852 10.1021/ac400503z

[b60] Ciobanasu C., Harms E., Tünnemann G., Cardoso M. C. & Kubitscheck U. Cell-penetrating HIV_1_ TAT peptides float on model lipid bilayers. Biochemistry 48, 4728–4737 (2009). 19400584 10.1021/bi900365s

