# Supplementary material for: Single Particle Dynamic Imaging and Fe3+ Sensing with Bright Carbon Dots Derived from Bovine Serum Albumin Proteins
Source: Sci Rep. 2015 Dec 4;5:17727. doi: 10.1038/srep17727 (PMC4669502; doi:10.1038/srep17727)
Supplement: Supplementary Information [file srep17727-s1.doc]

**Supplementary Information**

***for***

Single Particle Dynamic Imaging and Fe^3+^ Sensing with Bright Carbon Dots Derived from Bovine Serum Albumin Proteins

Qingxiu Yang, Lin Wei, Xuanfang Zheng, and Lehui Xiao*

Optical Imaging Laboratory, Key Laboratory of Chemical Biology & Traditional Chinese Medicine Research, Ministry of Education, Key Laboratory of Phytochemical R&D of Hunan Province, College of Chemistry and Chemical Engineering, Hunan Normal University, Changsha, Hunan, 410081, P. R. China.

**Supporting figures:**


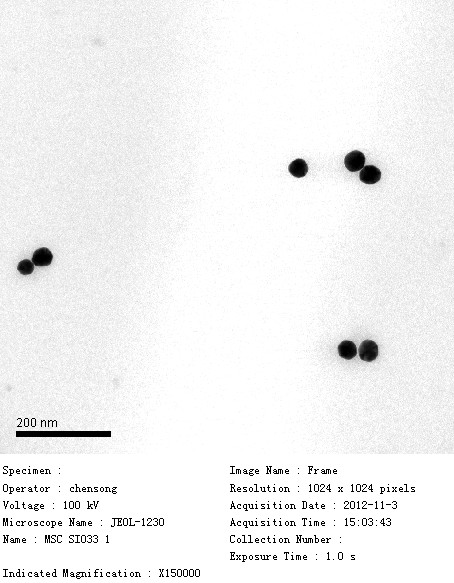


Figure S1. TEM image of BSA nanoparticles.


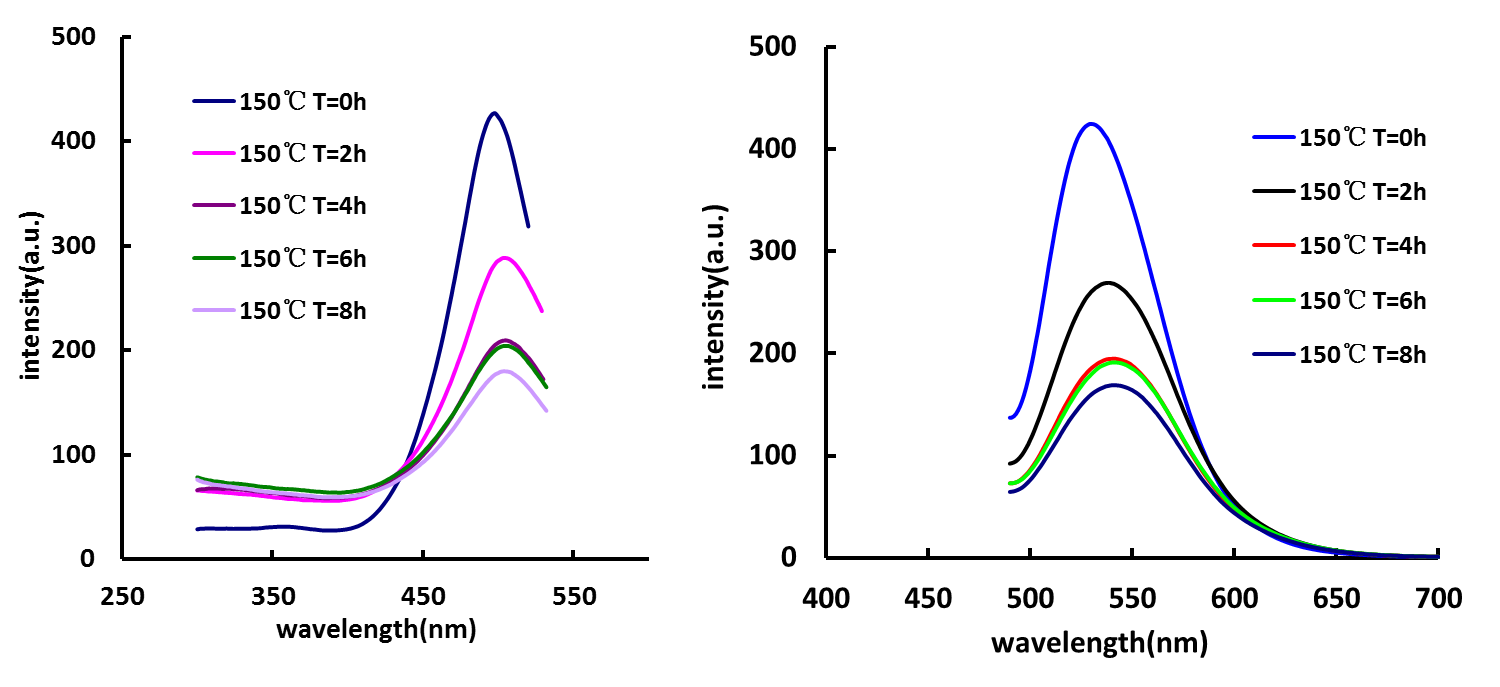


Figure S2. Fluorescence excitation (left) and emission spectra (right) of BSA nanoparticles after heated at 150 ^o^C for different times.


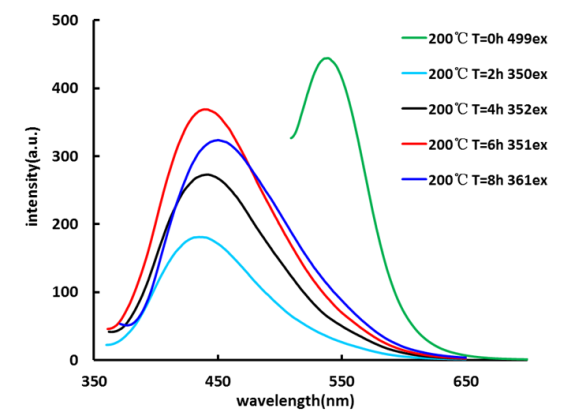


Figure S3. The fluorescence emission spectra of BSA nanoparticles after heated at 200 ^o^C for different times.


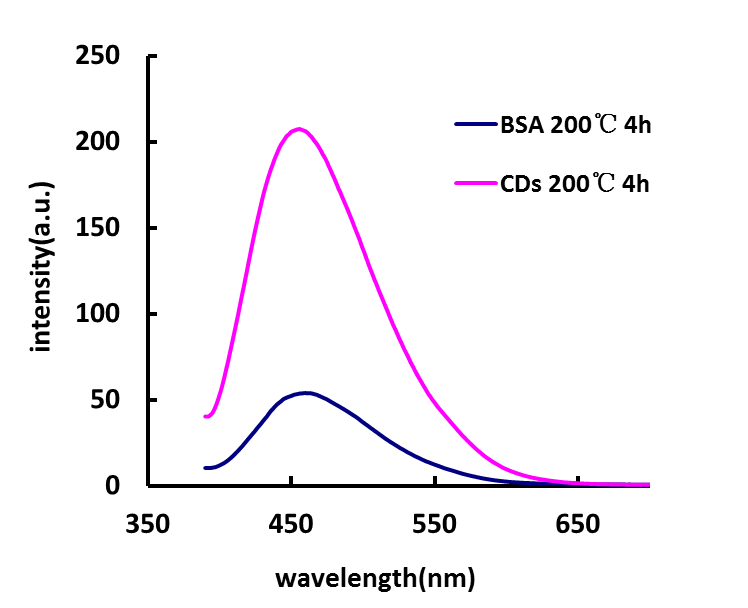


Figure S4. The fluorescence emission spectra of resultant Cdots from the precursor of BSA powder (blue) and BSA nanoparticles (red).


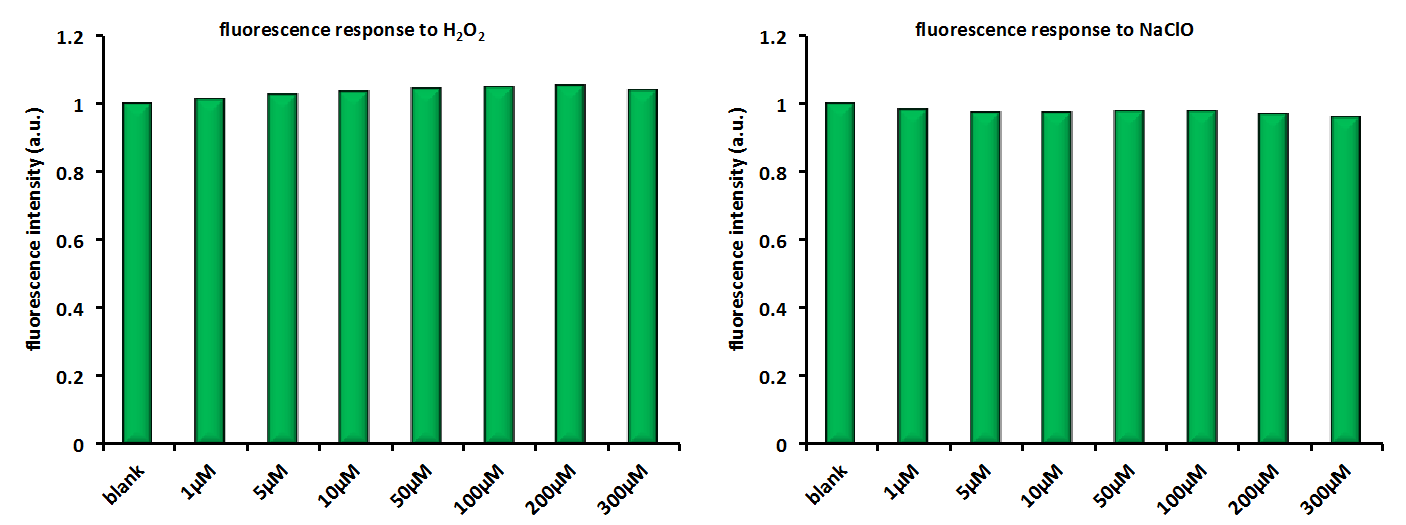


Figure S5. The fluorescence intensity of Cdots in H_2_O_2_ (left) and NaClO (right) solutions with different concentrations.

Figure S6. The fluorescence intensity of Cdots in DI water, NaCl, Na_2_CO_3_, and Na_3_PO_4_ solution.
